# Developing a Virus-Binding Bacterium Expressing Mx Protein on the Bacterial Surface to Prevent Grouper Nervous Necrosis Virus Infection

**DOI:** 10.4014/jmb.2103.03036

**Published:** 2021-06-14

**Authors:** Chia-Hua Lin, Jun-Jie Chen, Chiu-Min Cheng

**Affiliations:** 1 Ph.D. Program of Aquatic Science and Technology in Industry, College of Hydrosphere Science, National Kaohsiung University of Science and Technology, Kaohsiung City 80778, Taiwan; 2 Department and Graduate Institute of Aquaculture, National Kaohsiung University of Science and Technology, Kaohsiung City 80778, Taiwan

**Keywords:** Grouper nervous necrosis virus (GNNV), grouper Mx protein, bacterial surface expression, virusbinding bacterium

## Abstract

Grouper nervous necrosis virus (GNNV) infection causes mass grouper mortality, leading to substantial economic loss in Taiwan. Traditional methods of controlling GNNV infections involve the challenge of controlling disinfectant doses; low doses are ineffective, whereas high doses may cause environmental damage. Identifying potential methods to safely control GNNV infection to prevent viral outbreaks is essential. We engineered a virus-binding bacterium expressing a myxovirus resistance (Mx) protein on its surface for GNNV removal from phosphate-buffered saline (PBS), thus increasing the survival of grouper fin (GF-1) cells. We fused the grouper Mx protein (which recognizes and binds to the coat protein of GNNV) to the C-terminus of outer membrane lipoprotein A (lpp-Mx) and to the N-terminus of a bacterial autotransporter adhesin (Mx-AIDA); these constructs were expressed on the surfaces of *Escherichia coli* BL21 (BL21/lpp-Mx and BL21/Mx-AIDA). We examined bacterial surface expression capacity and GNNV binding activity through enzyme-linked immunosorbent assay; we also evaluated the GNNV removal efficacy of the bacteria and viral cytotoxicity after bacterial adsorption treatment. Although both constructs were successfully expressed, only BL21/lpp-Mx exhibited GNNV binding activity; BL21/lpp-Mx cells removed GNNV and protected GF-1 cells from GNNV infection more efficiently. Moreover, salinity affected the GNNV removal efficacy of BL21/lpp-Mx. Thus, our GNNV-binding bacterium is an efficient microparticle for removing GNNV from 10‰ brackish water and for preventing GNNV infection in groupers.

## Introduction

Viral nervous necrosis disease can cause mass mortality among groupers (*Epinephelus coioides*) and is a leading cause of economic loss in the aquaculture industry in Taiwan. Infected fish exhibit an abnormal swimming and swinging pattern and develop vacuoles within the brain and retina, ultimately leading to death. This pathology is caused by grouper nervous necrosis virus (GNNV), an RNA-containing piscine betanodavirus (*Nodaviridae*). It can spread through vertical transmission, contact with contaminated water, the food chain, or contact with contaminated aquacultural equipment. GNNV-infected fish constitute a major transmission vector for the disease [1-6]. Traditional methods of disease control in the aquaculture industry involve sterilization techniques, including ozone, ultraviolet radiation, iodine, and chlorine dioxide treatments [1, 4, 7-10]. However, at low doses, these treatments are ineffective in controlling GNNV infection; at relatively high doses, these treatments may harm surrounding aquatic animals, probiotics, and even humans. Therefore, developing new methods for safely removing GNNV from water and preventing further viral outbreaks is essential.

A method to bind and remove the virus from water could inhibit virus spread. Virus-binding proteins (VBPs) can efficiently recognize and bind to viruses, consequently inhibiting virus–host cell interactions and even viral replication. These proteins are present on the cell surface and are associated with channels through which the virus enters the cell, such as severe-acute respiratory syndrome-coronavirus (SARS-CoV) receptor angiotensin-converting enzyme 2 (ACE2) [11]. VBP production may be induced by adaptive immune responses against viral infections, such as responses involving therapeutic neutralizing antibodies (NAbs) against SARS-CoV-2 [13], or involved in innate immune responses against viral infection, such as those involving high-mannose-binding lectins that bind to the HA protein of an influenza virus and block its entry into the host cells [15].

Myxovirus resistance (Mx) proteins are interferon-induced antiviral factors that inhibit the replication of numerous RNA viruses [17, 18]. Chen *et al*. demonstrated that the effector domain of grouper Mx (gMx) proteins binds to the coat protein of nodavirus [19], indicating that gMx proteins are VBPs. Thus, VBPs have potential applications in the prevention and treatment of viral infections. Furthermore, because bacterial expression systems can be used to present various functional proteins on cell surfaces, bacterial lipoprotein-outer membrane protein A (lpp-ompA) and adhesin involved in diffuse adherence (AIDA) are commonly used for protein expression on the cell surface. VBPs may be engineered into bacterial surface expression systems for various purposes, including antigens as vaccines [20, 21], single-chain antibodies as diagnostic devices [22, 23], and enzymes as whole-cell biocatalysts [24, 25]. On the basis of these observations, we developed a virus-binding bacterium as a microparticle for Mx protein expression on the bacterial surface to reduce the GNNV load in aquatic environments and thus mitigate GNNV-associated mortality in aquaculture.

## Materials and Methods

### Bacteria, GNNV, and Cell Lines

We used *Escherichia coli* BL21 [F-ompT hsdSB (rB-, mB-) gal dcm (DE3), Novagen, USA] as the bacterial strain for this study. GNNV specimens were kindly gifted by Dr. Ching Long Huang (National Kaohsiung Marine University, Taiwan) and were grown in grouper fin (GF-1) cells (BCRC 960094). The GF-1 cells—which were fibroblast-like cells—were grown with some epithelioid histiocytes and maintained in L-15 medium (Sigma, USA) supplemented with 5% heat-inactivated bovine serum, 100 units/ml penicillin, and 100 units/ml streptomycin (Gibco Laboratories, USA) in humidified air at 28°C; they have been subcultured more than 200 times since 1995 [26].

### Membrane Mx Protein Gene Construction

**Fig. 1:**
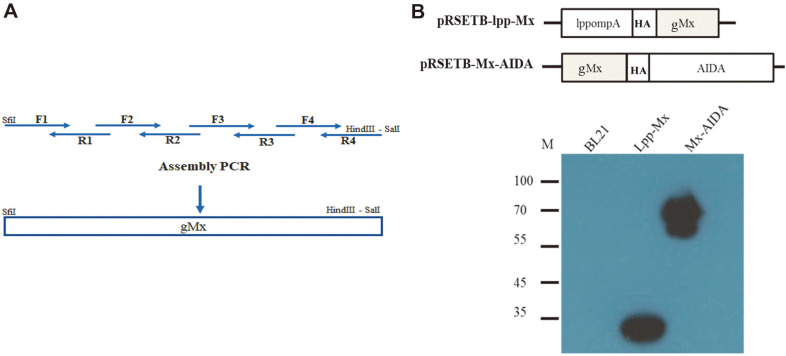
Construction and expression of surface Mx protein. (**A**) pRSETB-lpp-Mx and pRSETB-Mx-AIDA gene construction. (**B**) pRSETB-lpp-Mx and pRSETB-Mx-AIDA plasmids were transformed into BL21 cells to form BL21/lpp-Mx and BL21/Mx-AIDA, respectively. The expression of gMx was confirmed using western blotting with an anti-HA antibody. (HA was HA tag). Lane 1: BL21 cells as negative control; lane 2: BL21/lpp-Mx cells; lane 3: BL21/Mx-AIDA cells.

We cloned a truncated Mx gene (557–626 a.a.) based on the gMx DNA sequence [19, 25] by using the polymerase chain reaction (PCR) with the following primers: MxF1, 5′- GGCCCAGCCGGCCCTGGCGGA CCAGATCCCGCTGGTGATCCGC-3′, MxR1, 5′- GGGCCGCAGACTCCATGCAACATCTGGTAGCGG ATCACCAG-3′, MxF2, 5′- AGTCTGCGGCCCAGCTGCAGAGGGAGATGCTGCAGATGCTTC -3′, MxR2, 5′-TCAGCAAGAAACTCCATGTTCTCCTTGTCCTGAAGCATCTGCA-3′, MxF3, 5′- ATTTCTTGCTGA AGGAGGATTGTGACATCGGCAGCAAGAGGG -3′, MxR3, 5′-AGGCGTTTAGAGGCGACTCTGCAGACC AGCCCTCTTGCTGC-3′, MxF4, 5′- AAACGCCTCATGAAGGCCCGCGCATACCTGGTGGAGTTCTAG -3′, MxR4, 5′-GTCGACAAGCTTCTAGAACTCCACCAGGTAT-3′. This cloning was conducted to introduce the unique restriction sites of SfiI into the 5′ end and those of SalI and HindIII into the 3′ end of the PCR products (Fig. 1A). The PCR fragments were then digested with the enzymes SfiI and HindIII and inserted into pRSETB-lpp-βG [27] , replacing the βG to generate pRSETB-lpp-Mx; digested with SfiI and SalI and inserted into pRSETB-mβG-AIDA [27], replacing the βG to form pRSETB-Mx-AIDA; or digested with SfiI and HindIII and inserted into pET22b-pelB-aEGFR [28], replacing the aEGFR to generate pET22b-pelB-Mx.

### Bacterial Membrane Mx Expression

Both pRSETB-Mx-AIDA and pRSETB-lpp-Mx plasmids were transformed into BL21 cells to establish BL21/Mx-AIDA and BL21/ lpp-Mx cell lines. Membrane Mx expression was detected through western blotting using a monoclonal mouse anti-HA antibody. For the western blotting, 100 μl of transformed BL21 cells was mixed with 20 μl of 6X reducing sample buffer (375 mM Tris-HCl, 50% glycerol (v/v), 9% SDS (w/v), 9% beta-mercaptoethanol (v/v) and 0.03% Bromophenol blue (w/v)). This mixture was then loaded onto a sodium dodecyl sulfate-polyacrylamide gel electrophoresis (SDS-PAGE) gel (3% stacking gel; 10% running gel). After electrophoresis, proteins were transferred from the SDS-PAGE gel onto nitrocellulose membranes (Hybond C-extra, Amersham, Buckinghamshire, England). The membranes were blocked with 5% nonfat milk in phosphate-buffered saline with 0.05% Tween (PBST) for 1 h. The blocked membranes were then incubated with mouse anti-HA antibody (0.1 μg/ml, diluted in 2% nonfat milk in PBST; MMS-101R, Covance, USA) at 25°C for 1 h. Subsequently, the membranes were washed three times with PBST and incubated with horseradish peroxidase–conjugated goat antimouse IgG Fc (0.1 μg/ml) (Jackson ImmunoResearch Europe Ltd., UK) for 45 min. After being washed with PBST, the membranes were developed using an ECL luminescence kit (Millipore, USA) and exposed to an X-ray film.

### Enzyme-Linked Immunosorbent Assay (ELISA) for Detection of Mx Expressed on Bacterial Surface

BL21/Mx-AIDA, BL21/lpp-Mx, and BL21 cells were grown until an absorbance of 0.35 was reached at 600 nm; the cells were induced with 0.2 mM IPTG, and bacteria (1 × 10^7^ CFU/50 μl/well) were then transferred to a 96-well microtiter plate in 0.1 M NaHCO_3_ (pH 8.3) at 4°C overnight. After the removal of any uncoated bacteria through extensive washing in PBS, the plates were blocked overnight with 2% bovine serum albumin at 4°C and then incubated with 1 μg/ml anti-HA tag antibody (MMS-101P, Covance) in dilution buffer (PBS containing 0.5%bovine serum albumin) at 25°C for 1 h. Subsequently, the plates were washed five times with PBS and then incubated in 0.5 μg/ml horseradish peroxidase–conjugated goat anti-mouse IgG Fc (Jackson ImmunoResearch Europe Ltd.) at 25°C for 1 h. The plates were washed as described previously, and bound peroxidase activity was measured by adding 150 μl of 2,2′-azinobis (3-ethylbenzthiazoline-6-sulfonic acid, ABTS, Sigma-Aldrich) to each well in the presence of 0.003% H_2_O_2_ at 25°C for 30 min. Color development was measured at 405 nm using a microplate reader.

### Virus Titration and Purification

The GF-1 cells were seeded into 96-well plates (1.5 × 10^3^ cells/well). After overnight incubation at 25°C, the cells were treated with 50 μl of 10-fold diluted GNNV (10^−1^–10^−9^) in L-15 for 30 min. After the removal of GNNV, 200 μl of L-15 containing 1% FBS was added, followed by incubation at 25°C. Five days after inoculation, TCID_50_ values were calculated using the Reed and Muench method [29]. To purify GNNV, the procedure described by Dr. Shieh [30] was adopted. In brief, the GF-1 cells were seeded into a 10-cm dish (at a density of 8 × 10^6^ cells) and incubated overnight until the cells reached 95% confluence; the cells were treated with 1.5 ml of GNNV for 2 h, and 4.5 ml of L-15 medium was added, followed by incubation for 72 h. When an 80%–90% cytopathic effect was observed, the GF-1 cells were scraped and centrifuged at 10,000 ×*g* for 30 min. The supernatant was collected, and a final concentration of 5% polyethylene glycol (PEG) 20000 and 2.2% NaCl were added; the mixture was incubated at 4°C for 4–6 h and centrifuged at 10,000 ×*g* for 1 h. The pellets were resuspended in PBS, and an equal volume of Freon 113 was added, with vigorous shaking. After centrifugation at 3000 ×*g* for 10 min, the aqueous phase was collected, and 10%–40% (w/w) CsCl density gradient centrifugation was performed. The visible GNNV band was collected and dialyzed in PBS and stored at −70°C.

### GNNV Binding Activity of BL21/lpp-Mx, BL21/Mx-AIDA, and BL21 Cells

BL21/Mx-AIDA, BL21/lpp-Mx, and BL21 bacteria were grown until an absorbance of 0.35 was reached at 600 nm. For the immunoprecipitation assay, bacteria (1 × 10^8^ CFU/100 μl/tube) were allowed to interact with 1 × 10^4^ and 1 × 10^3^ TCID_50_/ml GNNV at 25°C in a shaker at 20 rpm for 10 min. The mixtures were then centrifuged at 6,000 ×*g* for 5 min, and the supernatants were removed. After being extensively washed in PBS five times, the pellets were resuspended in 100 μl of 1X reducing dye. The GNNV bound by bacteria was detected through western blotting with the rabbit anti-fish NNV antibody. For enzyme-linked immunosorbent assay (ELISA), after bacteria (1 × 10^8^ CFU/50 μl/well) were coated and blocked in a 96-well microtiter plate, the plates were incubated with 1.6 × 10^4^ TCID_50_ GNNV. Various soluble Mx (sMx) proteins were then added and incubated at 25°C for 10 min. The plates were then washed with PBS five times; the rabbit anti-fish NNV antibody (Genesis, Taiwan) was then added, and the samples were incubated at 25°C for 1 h. After being washed with PBS five times, the plates were incubated with 0.5 μg/ml horseradish peroxidase–conjugated goat anti-rabbit IgG Fc (Jackson ImmunoResearch) at 25°C for 1 h. Subsequently, the plates were washed five times with PBS, and the measurement of bound peroxidase activity and color development followed the aforementioned ELISA procedures.

### Sensitivity Analysis for GNNV Detection

We transferred 1 × 10^8^ CFU/well of either GNNV-binding bacteria (BL21/lpp-Mx) or GNNV in a graded concentration series (18.56, 92.8, 464, 2,320, and 11,600 TCID_50_/ml) into 96-well microtiter plates before incubation at 4°C overnight for the GNNV capture ELISA or indirect ELISA plates, respectively. After the removal of any GNNV or uncoated bacteria through extensive washing with PBS, the plates were blocked overnight with 2% bovine serum albumin at 4°C. The graded GNNV samples were then transferred to a capture ELISA plate and incubated for 10 min at 25°C. Both the capture and indirect ELISA plates were washed five times with PBS, used to detect bound GNNV, and then incubated with rabbit anti-fish NNV antibody (Genesis) as the primary antibody and horseradish peroxidase–conjugated goat anti-rabbit IgG Fc (Jackson ImmunoResearch) as the secondary antibody. The bound peroxidase activity and color development were measured in accordance with the aforementioned ELISA procedures. The sensitivity levels of the BL2/lpp-Mx-based capture and indirect ELISA were estimated on the basis of the lowest detectable GNNV concentration [31].

### Effect of Salinity on GNNV Removal Efficacy of BL21/lpp-Mx Bacteria

To evaluate whether salinity affected the GNNV removal ability of the BL21/lpp-Mx cells, the 1 × 10^8^ CFU BL21 (as control) and BL21/lpp-Mx cells were allowed to interact with 1 × 10^4^ TCID_50_/ml GNNV at various salinity levels (5‰, 10‰, 20‰, and 35‰) at 25°C in a shaker at 20 rpm for 10 min. The mixtures were then centrifuged at 6,000 ×*g* for 5 min, and the supernatants were collected. For the BL21/lpp-Mx-based capture ELISA, 50 μl of supernatant per well was analyzed using the aforementioned BL21/lpp-Mx-based capture ELISA procedures. Residual GNNV in PBS was estimated using the following formula:

$${\text{\% residual GNNV = (A}}_{\text{treatment-}}{\text{/A}}_{\text{original}}\text{)×100,}$$

where A is the absorbance.

### GNNV Removal Efficacy for BL21 and BL21/lpp-Mx Bacteria

To test whether the BL21 and BL21/lpp-Mx cells could efficiently remove GNNV from PBS, cultures of BL21 and BL21/lpp-Mx cells were grown until an absorbance of 0.35 was reached at 600 nm. The bacterial cultures were then harvested and washed five times in PBS. BL21 (as control) and BL21/lpp-Mx cells were allowed to interact with 1 × 10^4^ TCID_50_/ml GNNV at 25°C in a shaker at 20 rpm for 0, 1, and 10 min. The mixtures were then centrifuged at 6,000 ×*g* for 5 min, and the supernatants were collected. For the virus titration assay, the supernatants were diluted 10-fold using the aforementioned virus titration procedures. For the BL21/lpp-Mx-based capture ELISA, 50 μl of the supernatant per well was analyzed using the aforementioned BL21/lpp-Mx-based capture ELISA procedures. Residual GNNV in PBS was estimated using the following formula:

$${\text{\% residual GNNV = (A}}_{\text{treatment-}}{\text{/A}}_{\text{original}}\text{)×100,}$$

where A is the absorbance.

### BL21- or BL21/lpp-Mx -Mediated Cytotoxicity in GF-1 Cells Infected with GNNV

In this study, 1 × 10^4^ TCID_50_/ml GNNV was incubated with the BL21/lpp-Mx cells, the BL21 cells, or PBS for 10 min. The supernatant containing residual GNNV in PBS was collected, filtered, and diluted in L-15 medium (10×, 100×, 1,000×, and 10,000× dilutions). Moreover, 1.5 × 10^3^ GF-1 cells/well were seeded into 96-well microtiter plates and grown overnight. These cells were then treated with 25 μl/well of L-15 medium and 25 μl/well of either the aforementioned supernatant (i.e., the supernatant that had been graded, diluted, and filtered) or PBS as a control for 1 h at 25°C; subsequently, the supernatant was removed and replaced with 200 μl/well of fresh L-15 medium supplemented with 2% FBS. After an additional 120 h of culture, cell viability was determined using the Cell Counting Kit-8 assay (Sigma-Aldrich). The results are expressed as the proportional reduction in absorbance relative to that of untreated cells, as defined by the following formula:

% cell viability = 100 × (sample absorbance − background absorbance)/(control absorbance − background absorbance).

### Statistical Analysis

Data were obtained from three independent experiments, and the final results are shown as mean ± SD. Student’s *t*-test was used to determine statistical significance. A *p*-value of<0.05 was considered significant. All statistical analyses were performed using GraphPad Prism 6.0.

## Results

### Expression of Mx on Bacterial Membranes

To develop bacteria for the surface expression of Mx, we constructed the Mx plasmids pRSETB-lpp-Mx and pRSETB-Mx-AIDA for Mx protein expression on bacterial surfaces by fusing the gene encoding Mx to the C-terminus of the bacterial lpp-ompA gene (lpp-Mx) or to the N-terminus of the bacterial autotransporter AIDA-I gene (Mx-AIDA) (Fig. 1A), and subsequently, both plasmids were transformed to establish BL21/lpp-Mx and BL21/Mx-AIDA cells, respectively. To confirm protein expression in the BL21/lpp-Mx and BL21/Mx-AIDA cells, these cells were grown and induced with IPTG; protein expression was detected through western blotting with the anti-HA antibody. As presented in Fig. 1B, the BL21/lpp-Mx and BL21/ Mx-AIDA cells expressed proteins with expected sizes of 30 and 70 kDa size, respectively.

### Surface Expression of Functional GNNV-Binding Proteins on BL21 Cells

**Fig. 2:**
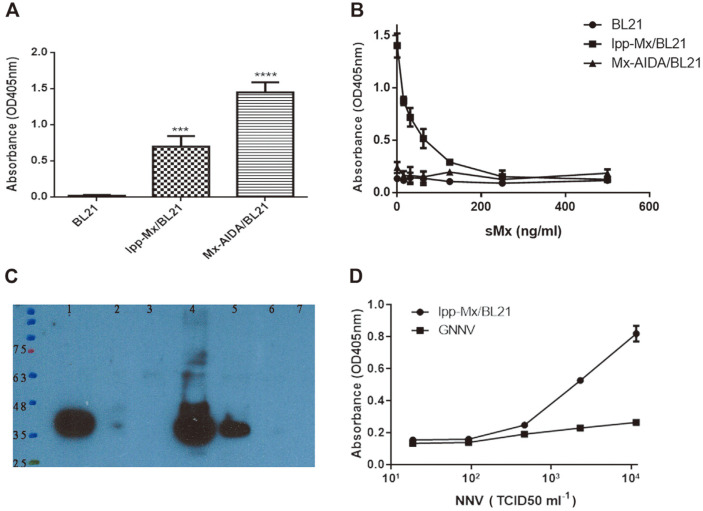
Surface expression of functional GNNV-binding proteins on bacteria. (**A**) Surface expression of Mx proteins on BL21 cells was analyzed through ELISA with the anti-HA antibody, and (**B**) GNNV binding activity was analyzed through capture ELISA after the addition of GNNV mixed with various sMx proteins and the rabbit anti-NNV antibody as the detection antibody. Absorbance was measured at 405 nm. Error bars indicate the standard deviation (SD) of three replicates. Statistical analysis was conducted using paired *t*-tests; *p < 0.05 compared with BL21 cells. (**C**) BL21, BL21/lpp-Mx, and BL21/ Mx-AIDA cells were incubated with GNNV for 10 min; after washing, GNNV in bacterial pellets was detected using western blotting with the rabbit anti-NNV antibody. Lane 1, GNNV solution as positive control; Lane 2, BL21 cells incubated with 10^4^ TCID_50_ GNNV; Lane 3, BL21 cells incubated with 10^3^ TCID_50_ GNNV; Lane 4, BL21/lpp-Mx cells incubated with 10^4^ TCID_50_ GNNV; Lane 5, BL21/lpp-Mx cells incubated with 10^3^ TCID_50_ GNNV; Lane 6, BL21/Mx-AIDA cells incubated with 104 TCID_50_ GNNV; Lane 7, BL21/Mx-AIDA cells incubated with 10^3^ TCID_50_ GNNV. (**D**) GNNV binding activity was measured in a 96- well plate coated with 1 × 10^8^ CFU/well of BL21/lpp-Mx bacteria (BL21/lpp-Mx-based capture ELISA) or in a series of GNNV dilutions (indirect ELISA). Binding activity was assessed at an absorbance of 405 nm. Error bars indicate the SD of three replicates.

To confirm whether the gMx protein was expressed on bacterial cell surfaces, we coated ELISA plates with the BL21, BL21/lpp-Mx, and BL21/Mx-AIDA cells and detected surface expression through ELISA with the anti-HA tag antibody. As illustrated in Fig. 2A, the absorbance (representing the bound antibody) measured in the wells coated with the BL21/lpp-Mx (0.70 ± 0.12) and BL21/Mx-AIDA (1.45 ± 0.12) cells was significantly higher than that measured in the wells coated with the BL21 cells (0.02 ± 0.01, *p* < 0.0001), indicating that lpp-ompA and AIDA could direct gMx to the bacterial surface. To verify whether the GNNV binding activity of the gMx fusion proteins on the bacterial surface was retained, the BL21, BL21/lpp-Mx, and BL21/Mx-AIDA cells were coated in 96-well microtiter plates as GNNV-binding microparticles. After the addition of GNNV mixed with various concentrations of sMx proteins, GNNV binding activity was detected through ELISA using the rabbit anti-NNV coat protein antibody. As displayed in Fig. 2B, the absorbance (representing the bound antibody) measured in the wells coated with BL21/lpp-Mx bacteria decreased as sMx protein concentration increased, but the absorbance for BL21 and BL21/Mx-AIDA bacteria was not affected by the sMx protein concentration. Furthermore, after the incubation of BL21, BL21/lpp-Mx, and BL21/Mx-AIDA bacteria with GNNV, the GNNV bound on the cells after washing with PBS was detected through western blotting using the anti-NNV antibody. As shown in Fig. 2C, the band was present for the BL21/lpp-Mx cells but absent for the BL21/Mx-AIDA and BL21 cells. These results indicate that the BL21/lpp-Mx cells expressed the gMx protein on bacterial cell surfaces and retained GNNV binding activity.

### Sensitivity Analysis for GNNV Detection with Indirect and Capture ELISA

To test whether BL21/lpp-Mx-based capture ELISA was more sensitive than indirect ELISA for GNNV detection, 96-well microtiter plates were coated with BL21/lpp-Mx bacteria or graded GNNV (18.56, 92.8, 464, 2,320, and 11,600 TCID_50_/ml). We measured the absorbance of the graded GNNV series after the rabbit anti-fish NNV antibody interacted with horseradish peroxidase–conjugated goat anti-rabbit IgG. Absorbance increased in a GNNV concentration-dependent manner for both types of ELISA (Fig. 2D). However, the absorbance measured for the capture ELISA performed using the BL21/lpp-Mx cells (0.818 ± 0.03) was three times higher than that measured for the indirect ELISA (0.264 ± 0.01) at a GNNV concentration of 1.6 × 10^4^ TCID_50_/ml. Additionally, the absorbance measured for the capture ELISA performed using the BL21/lpp-Mx cells (0.25 ± 0.006) was 1.3 times higher than that measured for the indirect ELISA (0.19 ± 0.008) at a GNNV concentration of 4.6 × 10^2^ TCID_50_/ml. The sensitivity of the BL21/lpp-Mx-based capture ELISA (121.56 TCID_50_/ml) was twice as high as that of the indirect ELISA (246.66 TCID_50_/ml). These results indicate that the BL21/lpp-Mx-based capture ELISA was more sensitive than the indirect ELISA in detecting GNNV.

### Effect of Salinity on the GNNV Removal Efficacy of BL21/lpp-Mx Bacteria

**Fig. 3:**
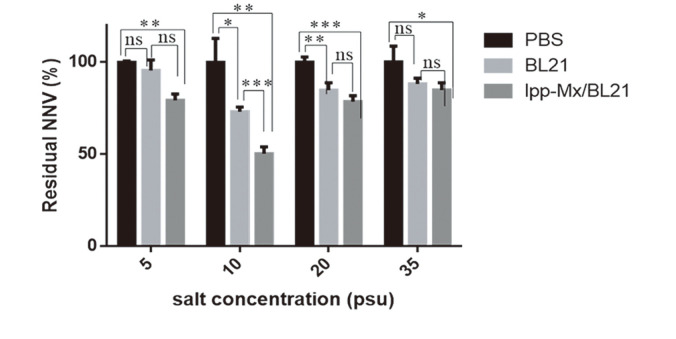
Effect of salinity on GNNV removal efficacy of BL21/lpp-Mx bacteria. Detection of residual GNNV in viral solution after incubation with BL21/lpp-Mx bacteria, BL21, or PBS at various salinity levels. Error bars indicate the SD of three replicates. Statistical analysis was conducted using paired t tests; *p < 0.05 compared with PBS treatment group.

To examine whether salinity affected the GNNV-binding ability of BL21/lpp-Mx, BL21, PBS (as control), and BL21/lpp-Mx were allowed to interact with GNNV at various salinity levels (5‰, 10‰, 20‰, and 35‰); residual GNNV in the solution was detected through BL21/lpp-Mx-based capture ELISA. The residual GNNV levels after the interaction of GNNV with BL21/lpp-Mx and BL21 bacteria at 10‰ salinity were 50.35% ± 3.68% and 73.11%± 2.52%, respectively, and those after the same interaction at 20‰ salinity were 78.66% ± 3.20% and 84.84% ± 4.01%, respectively (Fig. 3); these levels were significantly lower than those in PBS (*p* < 0.01). However, the residual GNNV levels at 5‰ were 95.55% ± 5.75% and 79.32% ± 3.38%, respectively, and at 35‰ were 88.22% ± 3.18% and 84.96% ± 3.93%, respectively. These levels were significantly lower than those in PBS (*p* < 0.05) but not lower than those in BL21 (*p* > 0.05). These results indicate that salinity affected the binding capacity of GNNV and BL21 bacteria, and the optimal GNNV capture ability was observed at 10‰ salinity.

### GNNV Removal Efficacy of BL21/ lpp-Mx Cells

**Fig. 4:**
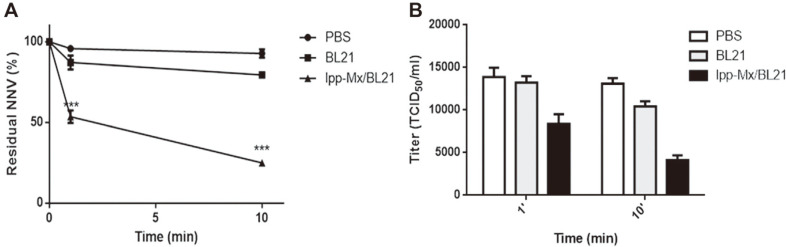
GNNV removal efficacy of BL21/lpp-Mxbacteria. Detection of residual GNNV in PBS after incubation with BL21/lpp-Mx bacteria, BL21 bacteria, or PBS through (**A**) BL21/lpp-Mx-based capture ELISA or (**B**) virus titration. The bar represents the SD of three replicates. Statistical analysis was conducted using the paired t-test; *p < 0.05 compared with PBS treatment group (NNV).

To test whether the BL21/lpp-Mx cells effectively removed GNNV from the solution, 1.6 × 10^4^ TCID_50_/ml GNNV was incubated with the BL21/lpp-Mx cells, BL21 cells, or PBS for 0, 1, and 10 min; residual GNNV in PBS was detected through the BL21/lpp-Mx-based capture ELISA or virus titration. Residual GNNV in PBS decreased with time when the viral solution was incubated with BL21/lpp-Mx and BL21 bacteria (Fig. 4). After 1 min of incubation, the residual GNNV level in the supernatant with the BL21/lpp-Mx cells (53.51% ± 3.84%) was significantly lower than those in the supernatant with the BL21 cells (88.27% ± 5.40%) and PBS (95.79% ± 1.16%; *p* < 0.05). After 10 min of incubation, the residual GNNV level in the BL21/ lpp-Mx cells (24.88% ± 0.92%) decreased more significantly than did those in the BL21 cells (79.40% ± 1.56%) and PBS (92.31% ± 2.61%; *p* < 0.001). In addition, the viral titer of residual GNNV was examined. As presented in Fig. 4B, the residual GNNV level in the supernatant with the BL21/lpp-Mx cells was 1.66 times lower than that in PBS and 1.58 times lower than that in the BL21 cells after 1 min of incubation; the residual GNNV level in the supernatant with the BL21/lpp-Mx cells was 3.16 times lower than that in PBS and 2.51 times lower than that in the supernatant with the BL21 cells after 10 min of incubation. These results indicate that the BL21/ lpp-Mx cells could bind to GNNV in PBS solution, and the degree of binding increased with time. However, the BL21/ lpp-Mx cells could more rapidly bind to and remove GNNV from the solution compared with the BL21 cells.

### BL21/lpp-Mx -Mediated Cytotoxicity in GF-1 Cells Infected with GNNV

**Fig. 5:**
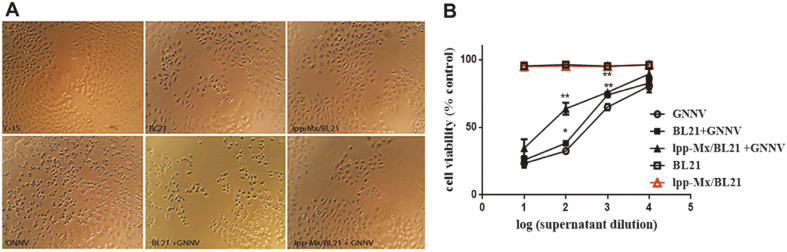
Cytotoxicity of BL21 and BL21/lpp-Mx for GF-1 cells infected by GNNV. BL21/lpp-Mx cells, BL21 cells, or PBS was incubated with GNNV for 10 min or not incubated. Residual GNNV-containing supernatants were filtered, diluted, and added to GF-1 cells. Cell morphology was observed using (**A**) phase contrast microscopy, and (**B**) cell viability was determined using the Cell Counting Kit-8 assay. Error bars indicate the SD of three replicates. Statistical analysis was conducted using paired t tests; *p < 0.05 compared with PBS treatment.

We tested whether the BL21/lpp-Mx cells efficiently removed GNNV from PBS and consequently reduced GNNV infection–induced cell death. Residual GNNV in the supernatant obtained from incubation with the BL21/lpp-Mx cells, BL21 cells, or PBS was filtered, diluted, and then added to the GF-1 cells. Cell morphology was observed under a microscope, after which cell viability was estimated. As displayed in Fig. 5A, the cell morphology observed after incubation with the supernatant from the BL21/lpp-Mx and BL21 bacterial treatment was similar to the normal cell morphology. Fig. 5B shows no cytotoxic effect. However, the cell morphology changed significantly and was similar to that of the cells incubated with the GNNV-containing supernatant from the BL21 bacterial treatment but was not similar to that of the cells incubated with the GNNV-containing supernatant from BL21/lpp-Mx bacterial treatment (Fig. 5A). Moreover, as presented in Fig. 5B, in the 100× diluted supernatant, the viability of the GF-1 cells treated with BL21/lpp-Mx bacteria (63.71 ± 4.32) was significantly higher than that of the cells treated with BL21 bacteria (38.24 ± 1.63, *p* = 0.0007) and PBS (32.52 ± 1.75, *p* = 0.0003), but the viability of the BL21-treated GF-1 cells was significantly higher than that of the PBS-treated GF-1 cells (*p* = 0.0143). In the 1000× diluted supernatant, cell viability after the BL21/ lpp-Mx treatment (76.0 ± 1.63) and BL21 treatment (74.0± 2.12) was significantly higher than that after PBS control treatment (64.9 ± 2.36, *p* = 0.0079 and 0.0026, respectively). No statistically significant difference was noted between the groups at 10× and 10000× dilutions. These results indicate that the supernatant from the BL21/lpp-Mx and BL21 treatments had no cytotoxic effect, and the expression of gMx on the BL21/lpp-Mx bacterial surface effectively removed GNNV from the solution and reduced GNNV infection–induced cell death.

## Discussion

Mx proteins have been used as hallmarks of interferon production and viral infection in vertebrates [17, 32, 33]. They have broad antiviral activity, such as prevention of viral RNA synthesis and protein expression, and they can inhibit the replication of many types of viruses, including Chinese giant salamander iridovirus, hepatitis B virus, reoviruses, and nodavirus [34-38]. Chen *et al*. demonstrated that gMx proteins could inhibit the synthesis of nodavirus RNA-dependent RNA polymerase, bind to the nodavirus coat protein, and inhibit viral replication. They reported that the N-terminal amino acid position 168 of the NNV coat protein could interact with the effector domain (335–626) of gMx. The minimal domain of gMx that binds to the NNV coat protein is GTP-CID (556–626 a.a.) [39], implying that this gMx region is a VBP for GNNV. In the present study, we cloned amino acids at the 556–626 position of gMx, fused the protein with a bacterial surface expression system in *E. coli* BL21 cells, and constructed a GNNV-binding bacterium. Our results demonstrate that the BL21/lpp-Mx bacteria expressed gMx on the cell surface and were effective at binding GNNV from the viral solution.

Bacterial surface expression systems have been used to express various heterologous proteins (*e.g.*, ScFvs [22, 23], enzymes [40, 41], viral antigens [20, 21], lipoproteins [42], and other foreign epitopes [43]) on bacterial cell surfaces. Outer membrane proteins are generally used as carriers for the expression of proteins on the surface of gram-negative bacteria. The lpp-ompA protein, which contains the first nine amino acids of the *E. coli* lipoprotein (signal sequence) and amino acids 46–159 of *E. coli* outer membrane protein A (transmembrane region), was the first protein used to successfully express full-length heterologous proteins on gram-negative bacterial surfaces [44]. Passenger proteins can be fused to the C-terminal of the lpp-ompA protein, and this configuration has been used to express several proteins (including single-chain antibodies, antigenic peptides, and enzymes) on the external surface of *E. coli* [45-47]. This has also been performed for the AIDA protein, which possesses an N-terminal signal peptide and a C-terminal membrane-spanning domain. Passenger proteins (*e.g.*, dimeric adrenodoxin [48], beta-glucuronidase [27], and antigenic proteins [49]) can be fused between the signal peptide and membrane-spanning domain and expressed on bacterial surfaces. In this study, we exploited these capabilities to engineer two bacterial cell lines, namely BL21/ lpp-Mx and BL21/Mx-AIDA, to bind free GNNV particles in solution. However, only the BL21/lpp-Mx bacteria exhibited GNNV binding activity. The BL21/lpp-Mx construct allows the N-terminus of gMx protein to be anchored to the bacterial surface through fusion with C-terminus of lpp-OmpA, and the C-terminus of the gMx protein is oriented away from the bacterial surface; moreover, the BL21/Mx-AIDA construct orients the N-terminus of gMx protein away from the bacterial surface. The C-terminus of the gMx protein is important for GNNV binding. Therefore, we speculate that the BL21/lpp-Mx construct may have the correct orientation and spatial distribution to bind to GNNV. Our results also reveal a reduction in viral load and cytotoxicity to GF-1 cells after GNNV interaction with the BL21/lpp-Mx bacteria, which is similar to previous findings that *E. coli* expressing anti-HIV-1 ScFv VRC01 on lpp-ompA could adsorb HIV-1 and neutralize HIV-1-infected Cf2Th cells [50]. Our results indicate that a GNNV-binding bacterium (BL21/lpp-Mx) has high potential to remove GNNV from aquatic environments and reduce damage to host cellular tissue.

ELISA is an analytical antigen detection method that can be employed in aquaculture. Shieh *et al*. demonstrated that capture ELISA (using a mouse anti-GNNV monoclonal antibody for capture and a rabbit anti-GNNV polyclonal antibody for detection) represents a specific, sensitive tool for GNNV detection [30], but additional costs are incurred for capture antibodies. Furthermore, Hao *et al*. presented a poly-protein G-expressing bacterium (with the AIDA surface expression system) as an antibody-binding microparticle to enhance the sensitivity of immunoassays [51]. Our results reveal that BL21/lpp-Mx-based capture ELISA was more efficient than indirect ELISA at binding GNNV and that it might be a more cost-effective method for GNNV detection in aquaculture. Additionally, BL21/lpp-Mx bacteria can be used as a viral concentrator for GNNV detection in aquaculture; they can even be used for reducing environmental viral loads. Because numerous viruses can bind to Mx proteins, when the detection antibody is replaced with a specific antiviral antibody, our system can detect many types of viruses.

Salinity may influence protein–virus interactions. High salt concentrations may affect the number of salt bridges on a protein’s surface, thus affecting its stability [52]. A high salt concentration (>37 ppt) can cause the dissociation of VP15 and VP95 from the nucleocapsid of white spot syndrome virus (WSSV), affecting its assembly [6]. In addition, research has revealed that reducing the salinity of seawater increases the survival rate of groupers after betanodavirus infection [53], although we did not find any report confirming that low salinity reduces the binding force of NNV to cells. The present study demonstrated that the BL21/lpp-Mx bacteria had optimal removal efficacy at a salinity of 10‰, and higher (35‰) or lower (5‰) salinity reduced the GNNV removal efficacy. We speculate that salinity affected salt bridges on the protein’s surface, interfering with the binding of our bacteria to GNNV.

Despite these promising results, this study has some limitations. First, environmental salinity limited the GNNV removal efficacy of the BL21/lpp-Mx bacteria, potentially limiting their application in settings in which salinity levels cannot be controlled. Second, BL21 is not a probiotic that is suitable for aquaculture; it may harm the environment and ecology. Constructing the membrane-expressed Mx protein system on probiotics such as nitrobacteria may resolve the problem. Hence, through the use of nitrifying bacteria, which grow on the surfaces of biological filters presenting surface-expressed GNNV-binding proteins, GNNV-binding probiotics can be removed with the filters; this may alleviate the problem of continuous environmental release of GNNV and prevent future GNNV outbreaks. Furthermore, because Mx proteins can bind to numerous viruses, they can be used to remove different viruses and thereby create safer aquaculture environments.

In summary, we successfully expressed a functional GNNV-binding protein (gMx protein) on bacterial surfaces using a virus-binding microparticle. We fused the DNA sequence between the amino acid positions 556–626 of the Mx protein to the C-terminus of lpp-ompA. Thus, bacteria expressing this protein on their membrane (BL21/lpp-Mx bacteria) could rapidly bind to GNNV and efficiently remove the virus from the solution, thus protecting GF-1 cells from GNNV infection. Consequently, gMx expression on appropriate bacterial surfaces could help reduce environmental GNNV loads and prevent GNNV outbreaks in aquaculture.
